# Assisted Reproductive Techniques and submucous myoma

**DOI:** 10.5935/1518-0557.20210042

**Published:** 2021

**Authors:** Roshan Nikbakht, Parvin Dorfeshan

**Affiliations:** 1 Fertility Infertility Perinatology Research Center, Ahvaz Jundishapur University of Medical Science, Ahvaz, Iran; 2 Department of Social Medicine, Faculty of Medicine, Ahvaz Jundishapur University of Medical Sciences, Ahvaz, Iran

**Keywords:** infertility, myomas, assisted reproductive techniques (ART)

## Abstract

Submucous myomas have negative effects on fertility. To maintain fertility, conservative treatment should be suggested to women who wish to become pregnant, especially young patients. The patient was a 33-year-old woman, who had had secondary infertility for 3 years. Upon vaginal ultrasound, we noticed a submucous myoma measuring 26 mm x 31 mm with a compressive effect on the anterior surface of the endometrium. Ovarian reserve was low. The gold standard of myoma treatment is surgical intervention. But, for the following reasons: the adverse effects of surgery on the endometrium (intrauterine adhesion), the patient's refusal to undergo a myomectomy and her request for pregnancy, our strategy for treating was to reduce volume of submucous myoma and start the assisted reproductive techniques (ART) cycle, simultaneously. We administered three courses of Gonadotropin-releasing hormone analogues (GnRHa) and then induced controlled ovarian hyperstimulation. Ovum pick up was done. Finally, we transferred two embryos (4 and 6 cells). In subsequent patient visits, βhCG was positive after 14 days. At the last patient visit, the heart of the embryo was formed. From this finding, it may be concluded that combined GnRHa and ART is the treatment of choice for infertile women with uterine submucous myoma, considering the reduced ovarian reserve and response.

## INTRODUCTION

Uterine fibroids (or myomas) are benign tumors, associated with the smooth muscle wall of the uterus ^(Geethamala *et al*., 2016)^. Studies estimate that it occurs in 20-50% of women during their reproductive years ^(Zhang *et al*., 2010)^. Myomas can cause many disorders, including: pelvic pain, abnormal uterine bleeding, and fertility disorders ^(Sparic *et al*., 2016)^. Uterine fibroids are treated differently concerning their location and size. The various methods of treatment include medical treatment, open myomectomy, laparoscopic, and magnetic resonance-guided focused-ultrasound surgery ^(Van Heertum & Barmat, 2014)^. There is general agreement about the opposing effects of myomas on fertility. To maintain fertility, conservative treatment should be suggested to women who wishes to become pregnant, especially for young patients ^(Tinelli *et al*., 2018)^. Our goal with this case report is to discuss submucous myoma together with assisted reproductive techniques (ART) in a patient's pregnancy.

## CASE REPORT

The patient was a 33-year-old woman. She had been married for 10 years. She had no history of abortion and had a pregnancy that resulted in a 10-year-old girl. In her first pregnancy shed had had premature membrane rupture and had normal vaginal delivery. However, she had secondary infertility for 3 years. Therefore, she came to our fertility treatment clinic with a request for a new pregnancy on 7-29 of 2019.

The patient had regular menstruation cycles. Her past surgical history revealed that she had had a laparotomy due to ovarian cysts. In the initial examinations, there were no positive signs. In her vaginal ultrasound, we noticed a submucous myoma, measuring 26mm x 31mm with a compressive effect on the anterior surface endometrium. Her ovarian reserve (Anti-Mullerian Hormone and antral follicle counts) was low. The results of all systematic and ultrasound tests at the follow-up are summarized in Table 1.

**Table 1.** Summary of the laboratory tests report and ovarian ultrasonography.

**Test**	**^a^ BMI(kg/m^2^)**	**^b^ TSH(mIU/L)**	**^c^ PRL(ng/ml)**	**^d^ FSHIU/ml)**	**^e^ LHIU/ml)**	**^f^ AMH(mg/ml)**	**^g^ AFC (number)**	
							****Right ovary****	****Left ovary****
	37	1.25	8.2	3.8	1.8	0.4	2	4

a body mass index

b Thyroid Stimulating Hormone

c Prolactin

d Follicle-Stimulating Hormone

e Luteinizing hormone

f Anti-Mullerian Hormone

g Antral Follicle Counts

To maintain fertility in these cases, conservative treatment should be suggested to women who wish to become pregnant, especially young patients ^(Tinelli *et al*., 2018)^.

In this patient, due to the mass effect on the endometrium, initial treatment for pregnancy can be surgical. But, for the following reasons: the adverse effects of surgery on endometrium (intrauterine adhesion), the patient's refusal to undergo a myomectomy and her request for pregnancy, our strategy for treating this case was as follows.

Our treatment strategy for this patient was to reduce the volume of the submucous myoma and start the ART cycle. For this purpose, we administered three courses of Gonadotropin-releasing hormone analogues (GnRHa) (Dipherelin (triptorelin; Ipsen Pharma, Paris, France)) (three times every 28 days at a dose of 3.75 mg). After 14 days of the last injection (in vaginal ultrasound, the myoma had shrunk (17 mm), we induced controlled ovarian hyperstimulation with Gonal F (Merck Serono, Germany), Pergoveris (Merck Serono, UK) and Cetrotide (Cetrotide, Serono, Geneva, Switzerland). Then, we triggered ovulation with two subcutaneous Ovitrelle (250 µg, Merck Serono, Germany) vials. Then, 36 hours afterwards, we picked up the ovum. On the day of puncture, we obtained 5 oocytes (MII). We then performed intracytoplasmic sperm injection (ICSI) by fresh sperm on the same day. 48 hours after ICSI, we transferred two embryos (4 and 6 cells). In subsequent patient visits, βhCG was positive after 14 days. At the latter patient visit, the heart of the embryo was formed.

## DISCUSSION

There are different treatment options available to treat myoma complications. Treatment strategies are typically devised by considering the following: severity of symptoms, size and location of the myoma, the age of patient and the her desire for fertility ^(Viswanathan *et al*., 2007)^. Surgical intervention is the basic myoma treatment standard. Even though hysterectomy is a primary treatment, myomectomy is usually performed in women who wish to have a future fertility ^(Parker, 2007)^. However, it is not without the morbidities, and mortalities of any surgical methods ^(Donnez *et al*., 2003)^. As, uterine scars are associated with risks following: vicious placental implantation and uterine rupture ^(Rovio, 2008)^.

In this case, we encountered a submucous myoma (31x26 mm) accompanied by the compressive effect on the uterine endometrium. Given the effects and complications of myomectomy (e.g asherman's syndrome), and considering the reduced ovarian reserve and response, our strategy for treating infertility in this case were as follows:

1. Minimizing the size and volume of the submucosal myoma (by long acting GnRH agonist).
2. Performing the ART cycle followed by embryo transfer.

We used GnRHa to reduce the submucosal myoma volume and this is supported by "GnRHa can effectively reduce uterine myoma volume, reduce heavy menstrual bleeding, and restore hemoglobin levels by inducing an iatrogenic reversible menopause" ^(Deligdisch *et al*., 1997; Friedman *et al*., 1991; Khan *et al*., 2010; Palomba *et al*., 2002)^. According to studies, this process can be explained at the molecular level: GnRHa results in increased apoptosis, decreased angiogenesis and inflammatory responses in myoma lesions ^(Khan *et al*., 2010)^. So, the GnRH effect on its receptors, which have been identified in myomas and prevent their growth ^(Chen *et al*., 2005; Wiznitzer *et al*., 1988)^. This is supported by "those effects result in a 35% to 65% reduction in the myoma size along with the development of amenorrhea" ^(Witherspoon & Butler, 1934)^.

In this case, when the size of the submucous myoma was reduced (17 mm), we introduced the patient to the ART cycle. Currently, the patient is pregnant and, the volume of the submucous myoma has decreased to a minimum. Therefore, in submucous myomas, treatment strategies can be based on the volume and size of the myoma, demand and age of the patient to achieve the best possible outcomes.

## CONCLUSION

From these findings, we may conclude that combined GnRHa and ART is the treatment of choice for infertile women accompanied by uterine submucous myoma, considering the reduced ovarian reserve and their response.

## References

[r1] Chen W, Yoshida S, Ohara N, Matsuo H, Morizane M, Maruo T (2005). Gonadotropin-releasing hormone antagonist cetrorelix down-regulates proliferating cell nuclear antigen and epidermal growth factor expression and up-regulates apoptosis in association with enhanced poly(adenosine 5'-diphosphate-ribose) polymerase expression in cultured human leiomyoma cells. J Clin Endocrinol Metab.

[r2] Deligdisch L, Hirschmann S, Altchek A (1997). Pathologic changes in gonadotropin releasing hormone agonist analogue treated uterine leiomyomata. Fertil Steril.

[r3] Donnez J, Hervais Vivancos B, Kudela M, Audebert A, Jadoul P (2003). A randomized, placebo-controlled, dose-ranging trial comparing fulvestrant with goserelin in premenopausal patients with uterine fibroids awaiting hysterectomy. Fertil Steril.

[r4] Friedman AJ, Hoffman DI, Comite F, Browneller RW, Miller JD (1991). Treatment of leiomyomata uteri with leuprolide acetate depot: a double-blind, placebo-controlled, multicenter study. The Leuprolide Study Group. Obstet Gynecol.

[r5] Geethamala K, Murthy VS, Vani BR, Rao S (2016). Uterine Leiomyomas: An ENIGMA. J Midlife Health.

[r6] Khan KN, Kitajima M, Hiraki K, Fujishita A, Sekine I, Ishimaru T, Masuzaki H (2010). Changes in tissue inflammation, angiogenesis and apoptosis in endometriosis, adenomyosis and uterine myoma after GnRH agonist therapy. Hum Reprod.

[r7] Palomba S, Russo T, Orio F Jr, Tauchmanovà L, Zupi E, Panici PL, Nappi C, Colao A, Lombardi G, Zullo F (2002). Effectiveness of combined GnRH analogue plus raloxifene administration in the treatment of uterine leiomyomas: a prospective, randomized, single-blind, placebo-controlled clinical trial. Hum Reprod.

[r8] Parker WH (2007). Etiology, symptomatology, and diagnosis of uterine myomas. Fertil Steril.

[r9] Rovio P (2008). Uterine Leiomyomas-Studies on Etiology, Ultrasound Diagnostics and Surgical Treatment.

[r10] Sparic R, Mirkovic L, Malvasi A, Tinelli A (2016). Epidemiology of Uterine Myomas: A Review. Int J Fertil Steril.

[r11] Tinelli A, Kosmas I, Mynbaev OA, Favilli A, Gimbrizis G, Sparic R, Pellegrino M, Malvasi A (2018). Submucous Fibroids, Fertility, and Possible Correlation to Pseudocapsule Thickness in Reproductive Surgery. Biomed Res Int.

[r12] Van Heertum K, Barmat L (2014). Uterine fibroids associated with infertility. Womens Health (Lond).

[r13] Viswanathan M, Hartmann K, McKoy N, Stuart G, Rankins N, Thieda P, Lux LJ, Lohr KN (2007). Management of uterine fibroids: an update of the evidence. Evid Rep Technol Assess (Full Rep).

[r14] Witherspoon JT, Butler VW (1934). The etiology of uterine fibroids with special reference to the frequency of their occurrence in the Negro: an hypothesis. Surg Gynecol Obstet.

[r15] Wiznitzer A, Marbach M, Hazum E, Insler V, Sharoni Y, Levy J (1988). Gonadotropin-releasing hormone specific binding sites in uterine leiomyomata. Biochem Biophys Res Commun.

[r16] Zhang Y, Peng W, Clarke J, Liu Z (2010). Acupuncture for uterine fibroids. Cochrane Database Syst Rev.

